# Simultaneous multi-nuclide imaging via double-photon coincidence method with parallel hole collimators

**DOI:** 10.1038/s41598-021-92583-4

**Published:** 2021-06-25

**Authors:** Mizuki Uenomachi, Kenji Shimazoe, Kenichiro Ogane, Hiroyuki Takahashi

**Affiliations:** 1 Department of Nuclear Engineering and Management, School of Engineering, The University of Tokyo, 7-3-1, Hongo, Bunkyo-ku, Tokyo, Japan; 2 Department of Bioengineering, School of Engineering, The University of Tokyo, 7-3-1, Hongo, Bunkyo-ku, Tokyo, Japan; 3 JST, PRESTO, Saitama, 332-0012 Japan; 4 Department of Surgery, Graduate School of Medicine, The University of Tokyo, 7-3-1, Hongo, Bunkyo-ku, Tokyo, Japan; 5 Department of Nuclear Medicine, International University of Health and Welfare, 1-4-3, Minato-ku, Tokyo, Japan; 6 Institute of Engineering Innovation, School of Engineering, The University of Tokyo, 2-11-16, Yayoi, Bunkyo-ku, Tokyo, Japan

**Keywords:** Biomedical engineering, Imaging

## Abstract

Multi-tracer imaging can provide useful information in the definitive diagnosis and research of medical, biological, and pharmaceutical sciences. Single-photon emission computed tomography (SPECT) is one of the nuclear medicine imaging modalities widely used for diagnosis or medical research and has a multi-tracer imaging capability. One of the drawbacks of multi-tracer imaging is crosstalk from other gamma rays, which affects the reconstructed image. Scattering correction methods, such as the dual- and triple-energy window methods, are used for conventional SPECT imaging to reduce the background caused by the crosstalk. This study proposes another crosstalk reduction method. Some nuclides emit two or more gamma rays through intermediate levels. Thus, detecting these gamma rays with the coincidence method allows us to distinguish a true gamma ray signal and a background signal. The nuclide position can be estimated at the intersection of two gamma rays using collimators. We demonstrate herein simultaneous ^111^In and ^177^Lu imaging via the double-photon coincidence method using GAGG detectors and parallel hole collimators. The double-photon coincidence method greatly reduces the background caused by other gamma rays and offers higher-quality images than does conventional imaging.

## Introduction

In nuclear medicine, disease diagnosis and therapy are performed by using radiopharmaceuticals, which are specific ligands with radioisotopes. The radiopharmaceutical biodistribution reflects in vivo biological functional or metabolic information; thus, X-ray or gamma ray imaging is useful in lesion detection and disease diagnosis^1^. Conventionally, beta-emitters (e.g., ^131^I and ^90^Y) are widely used to treat malignant tumors^2–5^. Some therapeutic nuclides emit X-rays or gamma rays besides beta rays. The visualization of their biodistribution is helpful for evaluating its therapeutic effect. Multi-tracer imaging can provide useful information for the definitive diagnosis and research of medical, biological, and pharmaceutical sciences because the accumulation tendency of radiopharmaceuticals in the body depends on their kind. As imaging technologies, the single-photon emission computed technology (SPECT)^6,7^ and the positron emission tomography (PET)^8–10^ are the most common methods used in nuclear medicine. The PET can only visualize positron emitters, whereas the SPECT has a multi-tracer imaging capability with different single-photon emitters. In SPECT modalities, Pb-based collimators are equipped with energy-resolved detectors to determine the direction of incoming gamma rays. Dual-isotope SPECT imaging has been used for clinical applications^11–14^ and research of molecular imaging tracers^15–17^. Different tracers can be distinguished by detecting different energy photopeaks of gamma rays originating from each tracer. In principle, true and background signals cannot be distinguished only with energy information; thus, the background results in artifacts on the reconstructed image and quantification errors. When it comes to simultaneous multi-tracer imaging, the background signals include the crosstalk caused by Compton scattering and the overlap in the photopeak spectra originating from other nuclides. One of the approaches for crosstalk reduction is the usage of high-resolution-type SPECT scanners comprising semiconductor detectors^18^. CZT-based SPECT scanners were recently developed and can offer high-quality images because the energy resolution of CZT detectors is higher than that of scintillator detectors^19–21^. A high-energy resolution SPECT scanner can discriminate close energy peaks and reduce the crosstalk by a narrower energy window. Other approaches are scatter correction methods, such as the dual-energy window^22,23^ and triple-energy window^24,25^ subtraction methods.

**Figure 1:**
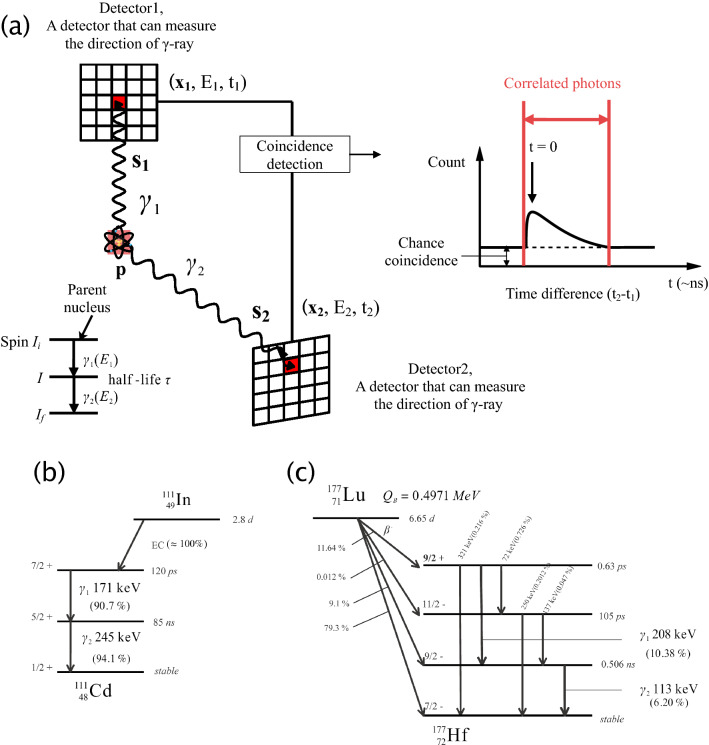
Principle of the double photon coincidence imaging and decay scheme of ^111^In and ^177^Lu. (**a**) The conceptual schematic of the double photon coincidence imaging. (**b**) ^111^In decay scheme. Two gamma rays with 171 and 245 keV energies are emitted through the intermediate level with 85 ns duration after electron capture. (**c**) ^177^Lu decay scheme. ^177^Lu emits beta rays and some gamma rays. Gamma rays with 208 and 113 keV energies are mainly emitted through the intermediate level with 0.506 ns duration. Figure (**a**) was created using the software (Wondershare EdrawMax, v.10.1.5, https://www.edrawsoft.com).

This study proposes a novel method using coincidence detection to reduce crosstalk and distinguish nuclides. Some nuclides emit two or more successive gamma rays, which are called cascade gamma-rays, through intermediate levels of atomic nucleus via nuclear spin. Thus, similar to PET imaging, which detects coincidence events of annihilation gamma rays, the background signals can be reduced to random events within a time window using coincidence detection and energy selection. Moreover, since there is the time and space correlation between the emission directions of cascade gamma-rays, the probability distribution of the nuclide with coincidence detection can be limited to the intersection of each distribution obtained by single-photon detection if the imaging system can determine the direction of incoming gamma-rays. Figure 1a show the conceptual schematic of the double-photon coincidence imaging we proposed in this paper, which utilizes the correlation of cascade gamma-rays originated from one atomic nucleus. A nuclide emits cascade gamma-rays (${\gamma }_{1}\,\text{and}\,{\gamma}_{2}$) with energies of E_1_ and E_2_ through the intermediate state with the half-life of $\tau$, and a detector can measure the energy E, detection position **x**, detection time t, and direction **s** (unit vector). When the cascade gamma-rays are detected with detector1 and detector2, the value of an image voxel $\lambda \left({\textbf{p}}_{i}^{\boldsymbol{\prime}}\right)$ to add is ideally like below:

1 $$\lambda \left( {{\mathbf{p}}_{i}^{\prime } } \right) = \frac{{f\left( {a_{i} } \right)}}{{\sum\nolimits_{{i = 0}}^{N} f \left( {a_{i} } \right)}}$$

2 $$f\left( {a_{i} } \right) = \left\{ {\begin{array}{*{20}c} {1{\text{~}}\left( {a_{i} = 0} \right)} \\ {0{\text{~}}\left( {a_{i} \ne 0} \right)} \\ \end{array} } \right.$$

3 $${a}_{i}=\left|\left({\textbf{x}}_{1}-{\textbf{p}}_{i}^{\boldsymbol{\prime}}\right)\times {\boldsymbol{s}}_{1}\right|+\left|\left({\textbf{x}}_{2}-{\textbf{p}}_{i}^{\boldsymbol{\prime}}\right)\times {\boldsymbol{s}}_{2}\right|$$

where ${\textbf{p}}_{i}^{\boldsymbol{\prime}}$ is the position of the voxel and *i* is the index corresponding to the image voxel number ($i=\text{0,1},2,\dots ,N)$. The correlated photons can be extracted by the coincidence detection of ${\gamma }_{1}\,\text{and}\,{\gamma }_{2}$ within a time window. When the time resolution of system is faster than the half-life $\tau$, the delay caused by the half-life can be measured in the spectrum of time difference.

For example, ^134^Cs mainly emits 796 and 605 keV gamma rays through the intermediate level with a 5.12 ps duration. We have already applied the double-photon coincidence method to Compton imaging and demonstrated its effectiveness in improving the signal-to-background ratio (SBR) by using the intersection of double Compton cones^26,27^. The Compton imaging results of ^134^Cs in the ^137^Cs background in Ref.^27^ showed the crosstalk reduction capability of the double-photon coincidence method. This method can also be applied to radionuclide imaging using a Pb-based collimator. The direction of incoming gamma rays is determined through mechanical collimation; thus, the coincidence detection of two photons can estimate the nuclide position in these intersection points. We have already demonstrated ^111^In imaging using two GAGG pixel detectors equipped with parallel hole collimators. The ^111^In position was correctly imaged by detecting coincidence events of 90°^28^. ^111^In is used for SPECT imaging and emits 171 and 245 keV gamma rays through the intermediate level with 85 ns duration (Fig. 1b). Its half-life is approximately 2.8 days. In these previous study^26–28^, the double photon coincidence method was applied to imaging only one double-photon emitter even though this method also has the potential to distinguish multiple double-photon emitting nuclides. Recently, we realized that a nuclide of ^177^Lu, which is used for treating neuroendocrine tumors^29–31^, was another double-photon emitter in nuclear medicine. ^177^Lu emits 208 and 113 keV gamma rays through the intermediate level with 0.506 ns duration after beta minus decay (Fig. 1c)^32^. Its half-life is approximately 6.65 days. The gamma rays from ^177^Lu are suited for SPECT imaging; thus, the ^111^In and ^177^Lu combination is also used for dual-isotope imaging as a tool in the molecular imaging tracer design^15^. We demonstrate herein the multiple double-photon emitting nuclides imaging of ^111^In and ^177^Lu via the double photon coincidence method for the first time by using four 8 $\times$ 8 array GAGG detectors equipped with 8 $\times$ 8 array parallel hole collimators in order to show the capability to distinguish nuclides and the effectiveness of the double-photon coincidence method for crosstalk reduction and SBR improvement.

## Results

### Simultaneous ^111^In and ^177^Lu imaging in microtubes

**Figure 2:**
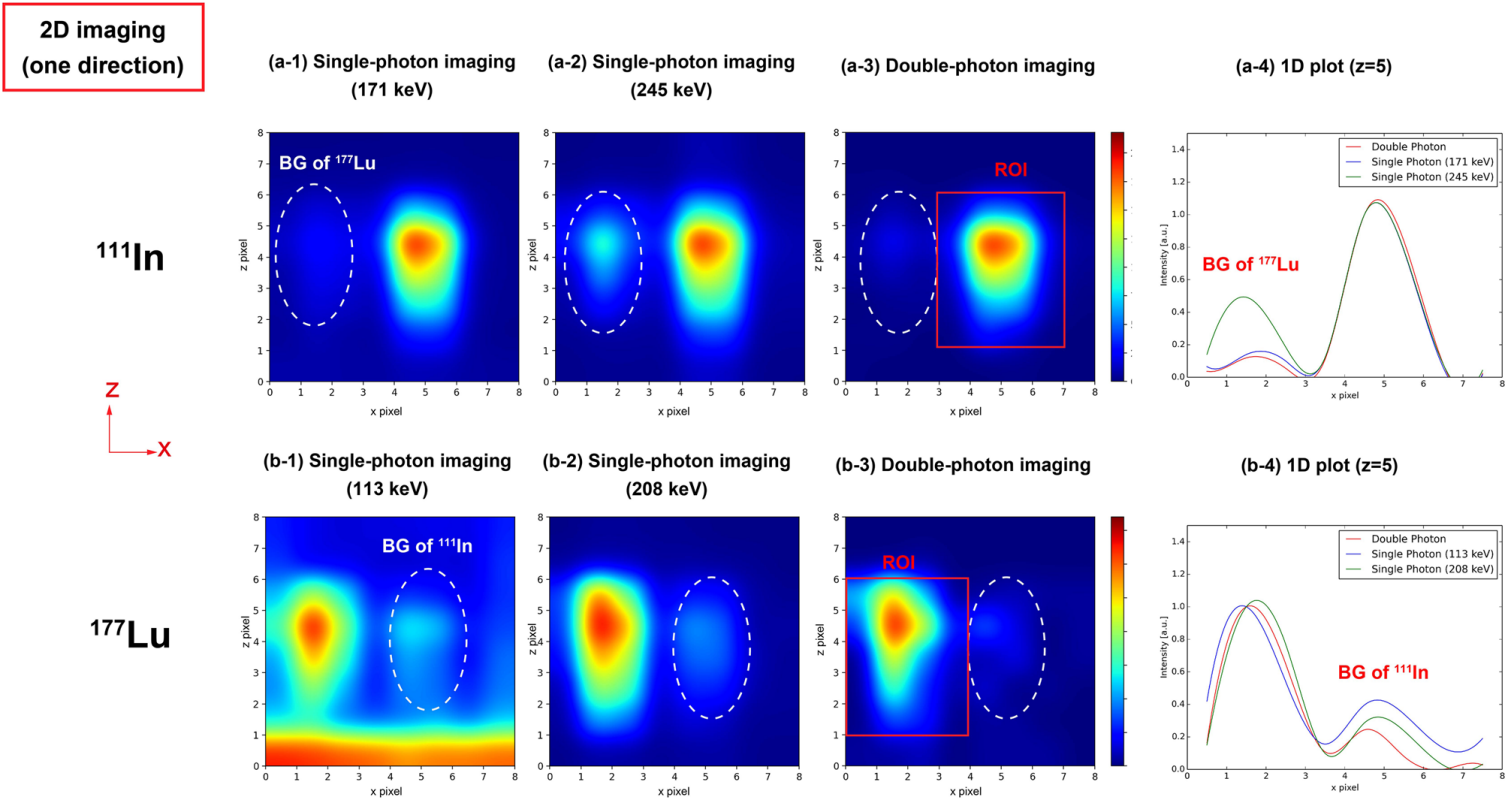
2D imaging results for ^111^In and ^177^Lu in the microtubes in one direction. (**a-1**) Single-photon imaging using 171 keV gamma rays. (**a-2**) Single-photon imaging using 245 keV gamma rays. The overlaps in the photopeak spectra of 208 and 245 keV mainly contribute to the background (BG) events that make the artifacts in the image. (**a-3**) Double-photon coincidence imaging. (**a-4**) 1D plot at the *z*-pixel = 5. The background in the ^177^Lu position is reduced by the coincidence method. (**b-1**) Single-photon imaging using 113 keV gamma rays. The scattering events of ^111^In mainly contribute to the BG events that make the artifacts in the image. (**b-2**) Single-photon imaging using 208 keV gamma rays. (**b-3**) Double-photon coincidence imaging. (**b-4**) 1D plot at the z-pixel = 5. The background in the ^111^In position is reduced by the coincidence method.

**Figure 3:**
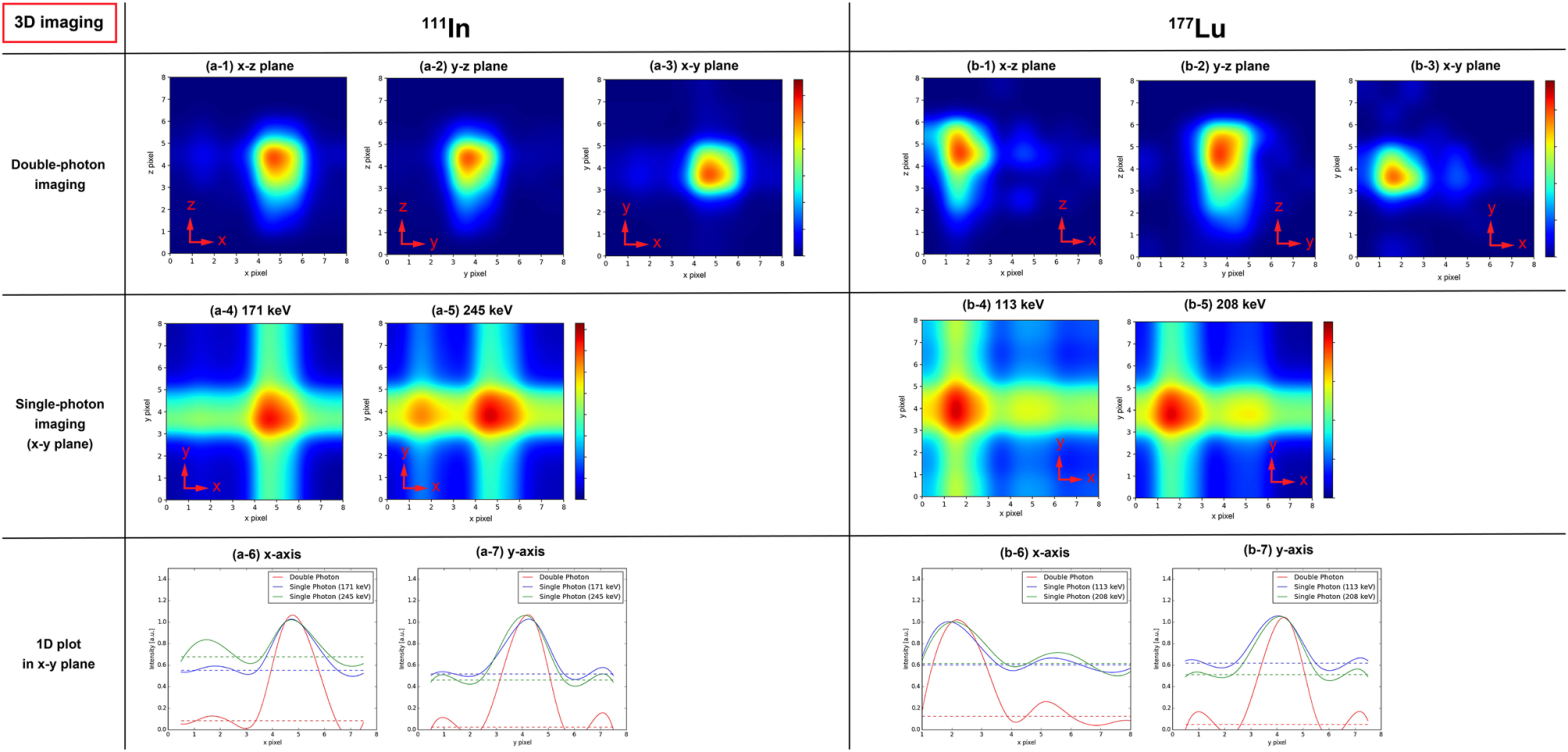
3D imaging results for ^111^In and ^177^Lu in the microtubes. (**a-1**) 2D slice images of ^111^In by double-photon imaging in the *x*–*z* plane (*y*-slice = 4). (**a-2**) *y*–*z* plane (*x*-slice = 3). (**a-3**) *x*–*y* plane (*z*-slice = 4). (**a-4**) 2D slice images of ^111^In by 171 keV single-photon imaging in the *x*–*z* plane (*y*-slice = 4). (**a-5**) 2D slice images of ^111^In by 245 keV single-photon imaging in the *x*–*z* plane (*y*-slice = 4). (**a-6**) 1D slice on the *x*-axis of the *x*–*y* plane (*y*-pixel = 4). (**a-7**) 1D plot in the *y*-axis of the *x*–*y* plane (*x*-pixel = 5). The dot lines denote the background value. (**b-1**) 2D slice images of ^177^Lu by double-photon imaging in the *x*–*z* plane (*y*-slice = 4). (**b-2**) *y*–*z* plane (*x*-slice = 3). (**b-3**) *x*–*y* plane (*z*-slice = 4). (**b-4**) 2D slice images of ^177^Lu by 113 keV single-photon imaging in the *x*–*z* plane (*y*-slice = 4). (**b-5**) 2D slice images of ^177^Lu by 208 keV single-photon imaging in the *x*–*z* plane (*y*-slice = 4). (**b-6**) 1D plot in the *x*-axis of the *x*–*y* plane (*y* pixel = 4). (**b-7**) 1D slice on the *y*-axis of the *x*–*y* plane (*x*-pixel = 1). The dot lines denote the background value.

**Figure 4:**
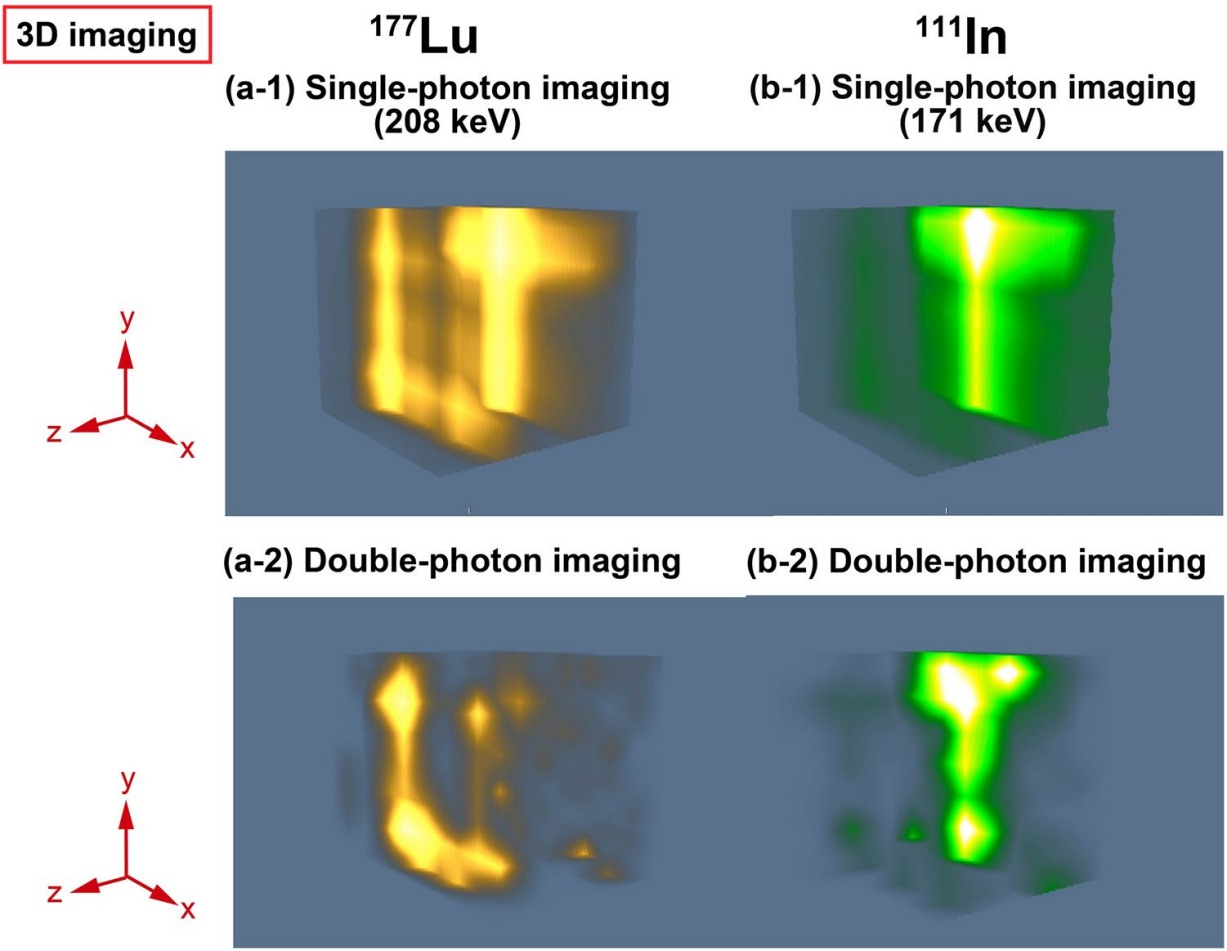
3D imaging results of the distribution sources. The RI source of ^111^InCl_3_ was in an acrylic case of the alphabetical letter “T,” while the RI source of ^177^LuCl_3_ was in an acrylic case of the alphabetical letter “U”. (**a-1**) 3D image of ^111^In by single-photon BP imaging using 171 keV gamma rays. (**a-2**) 3D image of ^111^In by double-photon coincidence imaging. (**b-1**) 3D image of ^177^Lu by single-photon BP imaging using 208 keV gamma rays. (**b-2**) 3D image of ^177^Lu by double-photon coincidence imaging.

Figure 2 shows the results of two-dimensional (2D) imaging in one direction, in which some artifacts are caused by the background events in images. Figure 2a-4, b-4 depict one-dimensional (1D) plots of the x-axis at the y-slice = 5. In both ^111^In and ^177^Lu images, we observed that the artifacts in the position of the other nuclides are reduced by using the double-photon coincidence method, although some remained. The artifacts in Fig. 2a-2 are mainly caused by the overlaps of the photopeak spectra of 208 and 245 keV. For the single-photon imaging of 113 keV gamma rays (Fig. 2b-1), scattering events with a collimator or a scintillator contribute to the artifact in the area of region of interest (ROI) of ^111^In. The artifact in the bottom line is assumed to be caused by scattering with the desk made of melanin. In this study, this artifact is not essential and can be solved by improving the experiment environment. Table 1 presents the SBRs in the images and the absolute detection efficiencies of Camera 1 in the ROI shown in Fig. 2a-3, b-3. Although the detection efficiencies of double-photon coincidence imaging decreased by approximately 10^−4^ times compared with those of single-photon imaging, the SBRs in the double-photon images increased by approximately two or more times (Table 1).

**Table 1:** SBR in images and absolute detection efficiency of Camera 1 for 2D imaging. The standard errors are also shown.

Nuclide	Imaging method	SBR in images	Absolute detection efficiency of Camera 1 in the ROI
^111^In	Double-photon imaging	9.31 $\pm$ 0.926	(4.22 $\pm$ 0.131) $\times$ 10^−8^
	Single-photon imaging (171 keV)	3.22 $\pm$ 0.003	(1.32 $\pm$ 0.001) $\times$ 10^−4^
	Single-photon imaging (245 keV)	2.01 $\pm$ 0.002	(8.17 $\pm$ 0.006) $\times$ 10^−5^
^177^Lu	Double-photon imaging	5.53 $\pm$ 1.001	(1.96 $\pm$ 0.139) $\times$ 10^−8^
	Single-photon imaging (113 keV)	0.60 $\pm$ 0.0004	(2.94 $\pm$ 0.002) $\times$ 10^−4^
	Single-photon imaging (208 keV)	2.77 $\pm$ 0.005	(8.09 $\pm$ 0.007) $\times$ 10^−5^

**Figure 5:**
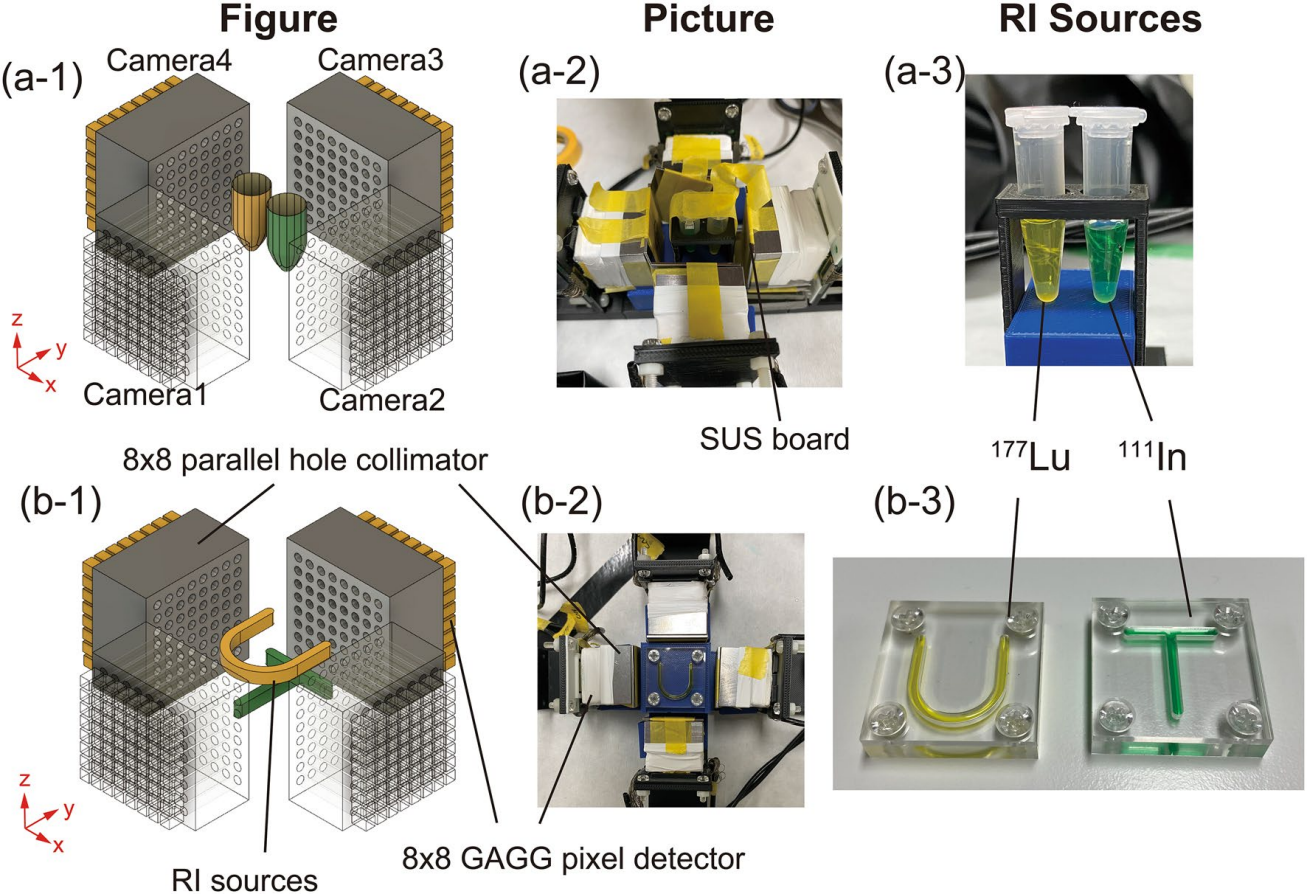
Experimental setup. (**a-1**–**a-3**) Experimental setup with the RI sources of ^111^In and ^177^Lu in 0.5 mL microtubes ((**a-1**) experimental setup, (**a-2**) experiment, and (**a-3**) RI sources in the microtubes). (**b-1**–**b-3**) Experimental setup with the RI sources of ^111^In and ^177^Lu in the acrylic cases of alphabetical letters “U” and “T” ((**b-1**) experiment setup, (**b-2**) experiment, and (**b-3**) RI sources in the acrylic cases of alphabetical letters “U” and “T”). Figure (**a-1**,**b-2**) were created using the software (Autodesk Fusion 360, v.2.0.9719, https://www.autodesk.com).

Figure 3 shows the results of three-dimensional (3D) imaging with four cameras. Specifically, Fig. 3a-4, a-5, b-4 and b-5 depict the 2D slice images in the *x*–*y* plane by the back projection (BP) method using single photons. Reconstruction methods, such as the filtered back projection and maximum likelihood–expectation maximization methods, were not applied herein; thus, intersection lines are drawn in single-photon images. Similar to 2D imaging in one direction, the artifacts caused by the background events from another nuclide can also be observed in (a-5), (b-4), and (b-5). By contrast, double-photon coincidence imaging can determine the RI source position at one voxel by taking the coincidence events at 90°. Figure 3a-3,b-3 illustrate the 2D slice images in the *x*–*y* plane of ^111^In and ^177^Lu by the double-photon coincidence method. Each position was correctly visualized. The 2D slice images in the *x*–*z* and *y*–*z* planes were also correctly obtained (Fig. 3a-1,a-2,b-1,b-2). Although the detection efficiencies of double-photon imaging were 10^4^ times smaller than those of single-photon imaging, the SBRs of the *x*- and *y*-axis of the *x*–*y* plane images were evaluated from the 1D plots in the *x*- and *y*-axis and found to greatly increase when the double-photon coincidence method was used (Table 2). The SBRs of the *y*-axis on the double-photon images were remarkably larger than those of the *x*-axis because the RI sources were placed along the *x*-axis, and some of the background in the ROI of another nuclide was not eliminated.

**Table 2:** Absolute detection efficiency for 3D imaging and SBR in the *x*- and *y*-axis.

Nuclide	Imaging method	SBR in the *x*-axis	SBR in the *y*-axis	Absolute detection efficiency of Camera 1 in the ROI
^111^In	Double-photon imaging	(11.65 $\pm$ 1.483)	(19.80 $\pm$ 2.277)	(4.38 $\pm$ 0.133) $\times$ 10^−8^
	Single-photon imaging (171 keV)	(1.81 $\pm$ 0.002)	(1.93 $\pm$ 0.003)	(5.14 $\pm$ 0.001) $\times$ 10^−4^
	Single-photon imaging (245 keV)	(1.48 $\pm$ 0.002)	(2.17 $\pm$ 0.003)	(3.94 $\pm$ 0.001) $\times$ 10^−4^
^177^Lu	Double-photon imaging	(8.00 $\pm$ 2.039)	(20.57 $\pm$ 8.159)	(2.51 $\pm$ 0.157) $\times$ 10^−8^
	Single-photon imaging (113 keV)	(1.66 $\pm$ 0.003)	(1.61 $\pm$ 0.002)	(1.19 $\pm$ 0.0003) $\times$ 10^−3^
	Single-photon imaging (208 keV)	(1.62 $\pm$ 0.003)	(1.96 $\pm$ 0.005)	(2.96 $\pm$ 0.001) $\times$ 10^−4^

### Simultaneous ^111^In and ^177^Lu imaging with distribution sources

Figure 4 shows the 3D images of the ^111^In and ^177^Lu distribution sources. ^111^InCl_3_ was in an acrylic case of the alphabetical letter “T”, whereas ^177^LuCl_3_ was in an acrylic case of the alphabetical letter “U”. The double-photon images in Fig. 4a-2,b-2 are clearly visualized without any rotation and reconstruction methods compared with the single-photon images in Fig. 4a-1,b-1. Although the detection sensitivities depending on voxels were not considered in this study, the sensitivity correction will improve the images of the distribution sources. In the single-photon image of 208 keV gamma rays from ^177^Lu (Fig. 4(b-1)), the background caused by ^111^In strongly contributed to the artifacts compared with that of the experiment using microtubes because the photopeaks of this experiment shifted from those of the calibration data due to the change of the gain of silicon photomultipliers (SiPMs) caused by the change of the environment temperature. This background was greatly reduced by the double coincidence method. The low intensity voxels in the alphabetical letter “U” were due to inhomogeneity of ^177^Lu solution volume. However, the artifacts were still seen in the double-photon image of ^177^Lu (Fig. 4b-2). More background can be reduced by implementing a temperature compensation circuit or by using a constant temperature chamber.

## Discussion

**Figure 6:**
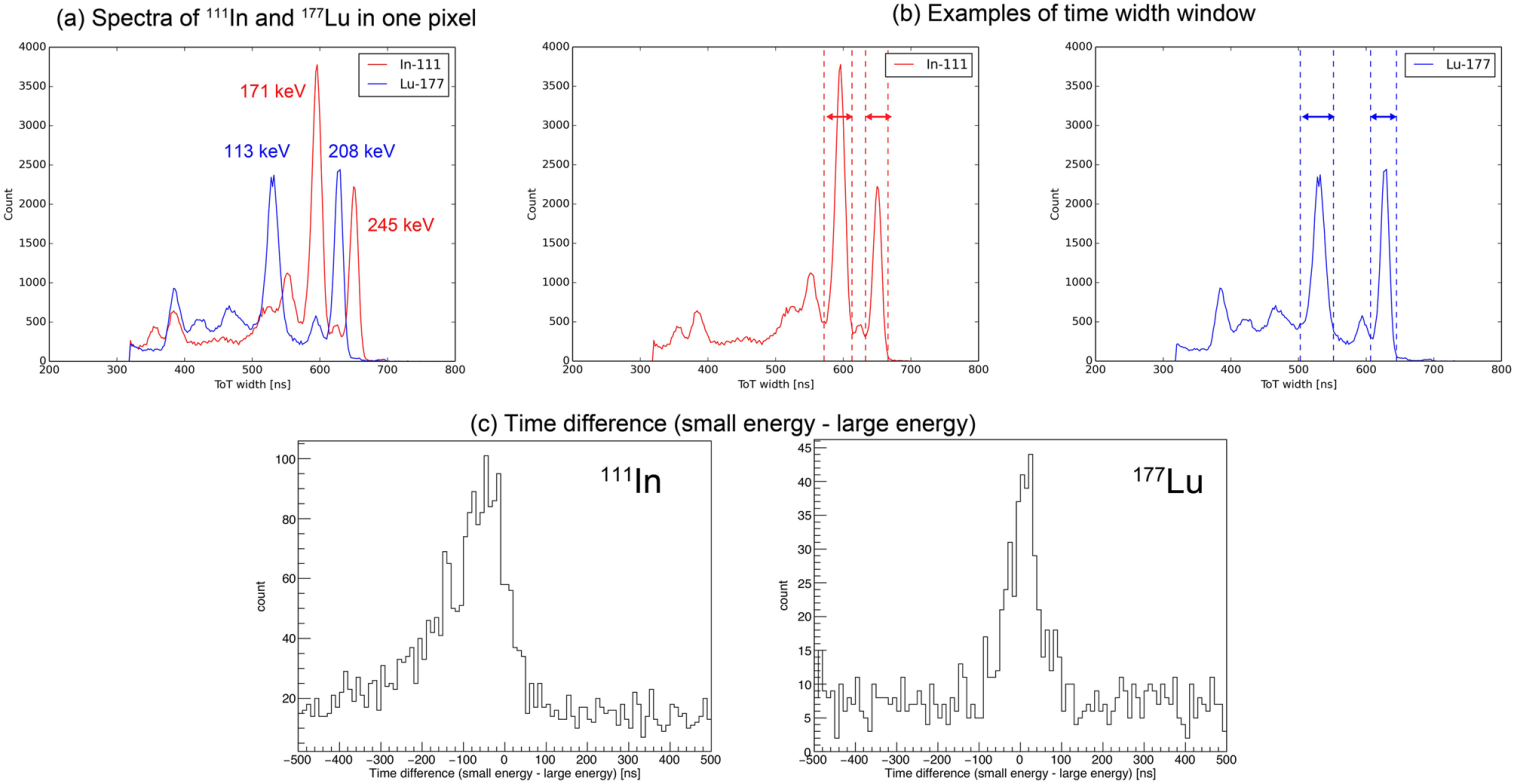
Spectra, examples of the time width window, and histogram of the time difference. (**a**) Spectra of ^111^In and ^177^Lu measured by 1 px of an HR-GAGG pixel detector. (**b**) Examples of the time width window for event selection. (**c**) Histograms of the time difference of ^111^In and ^177^Lu. A tail caused by the relatively long duration of 85 ns is shown in the ^111^In histogram.

We demonstrate herein simultaneous ^111^In and ^177^Lu double-photon coincidence imaging with an 8 $\times$ 8 array of parallel hole collimators. ^111^In and ^177^Lu, which are used in nuclear medicine, are double-photon emitters. ^111^In emits 171 and 245 keV gamma rays, whereas ^177^Lu emits 113 and 208 keV gamma rays. The energies of these gamma rays are close; thus, the crosstalk between the nuclides, including the overlaps in the spectra and scattering events, strongly affects the artifacts in the images with the system of ~ 12% energy resolution at 245 keV. Although the absolute detection efficiencies of double-photon imaging were 10^4^ smaller than those of single-photon imaging, the crosstalk was greatly reduced by double-photon coincidence imaging for both sources in the microtubes and distribution sources. However, some of the background was not eliminated. One of the causes is the shift of the peak positions caused by the changes in the SiPM gain due to the change of the environment temperature, especially for the long measurement experiment using the distribution sources. The energy windows were selected from the calibration data measured without a collimator; thus, the shift of the peak positions contributed to the background and resulted in incorrect coincidence events. Generally, the gain of SiPM increases as the temperature decreases^33^. The peak positions of 113, 171, 208, and 245 keV gamma-rays in the spectra of the measurement of distribution sources were shifted approximately 7.5% higher on average. The measurement of distribution sources was conducted during the night, thus, it was assumed that the temperature was lower than that in the measurement of calibration data. With the shift of peak positions, the overlap range in the time width window, which is corresponding to the energy window, of 208 keV gamma-ray with those of 171 and 245 keV gamma-rays was increased from 47.4 to 55.2%. Thus, the background of ^111^In appears in the single-photon image of the ^177^Lu distribution source (Fig. 4a-1) more than that of the micro tube (Fig. 3b-5). On the other hand, there was no overlap range in the 113 keV time width window with that of 171 keV gamma-ray, thus, incorrect events in the 113 keV window were just only scattering events of higher energies gamma-rays. While the detection probability of the incorrect event directly affects the background in the single-photon image, that of double photon coincidence method is greatly decreased because it is estimated as the product of the detection probabilities of the incorrect event in energy windows of gamma1 and gamma2. The peak shift will be solved by implementing a temperature compensation circuit or by using a constant temperature chamber. Another cause is the chance coincidence events within the time windows. The approximate ratios of the chance coincidence events to the coincidence events within the time windows are 35.4 $\pm$ 1.87% (^111^In) and 36.3 $\pm$ 2.56% (^177^Lu), which were calculated from the time difference histogram (Fig. 6c). Although it is impossible to completely eliminate the chance coincidence events, the time resolution improvement can reduce them. In this study, the sensitivity correction was not applied for imaging. The single-photon efficiency is inversely proportional to the square of the distance between a source position and a detector. Hence, the double-photon coincidence detection efficiency in a voxel is the product of detection efficiencies of the two detectors. The sensitivity correction of each voxel for the double photon coincidence imaging should be one of future works. There is room for improvement in terms of the time resolution of the detector system, experiment environment, and sensitivity correction, resulting in a higher-quality image.

In summary, the double-photon coincidence method is effective for multi-nuclide imaging to reduce the crosstalk between nuclides and distinguish them. Although the detection sensitivity of this method is significantly reduced compared with that of single-photon imaging, a higher-quality image can be offered without any rotation and reconstruction method by taking coincidence events, except at 180°. Recently, a new concept of the collimation method, which uses a scintillator detector as a collimator was proposed. For example, an active pinhole camera, which consists of a scintillator detector that has a pinhole and a non-hole detector, was reported^34^. The detection sensitivity of the double photon coincidence method with collimators we proposed in this paper can be improved by combing the new collimation method since the active collimator can detect gamma-rays.

## Methods

### Detector and collimator

We used a high-resolution-type Ce:Gd_3_Al_2.6_Ga_2.4_O_12_ (HR-GAGG) scintillator^35^. The characteristics of a GAGG scintillator are desirable energy resolution (4% with an avalanche photodiode [APD]), high light yield (56,000 photons/MeV), high density (6.63 g/cm^3^), moderate timing resolution (150 ns), non-deliquescence, and non-self-irradiation. The 8 $\times$ 8 array of HR-GAGG scintillators with a pixel size of 2.5 mm $\times$ 2.5 mm $\times$ 4 mm was used as the pixel detectors. The pitch size was 3.2 mm $\times$ 3.2 mm. Each crystal was separated by BaSO_4_ reflectors that can improve the light output compared with that with a Teflon tape or an enhanced specular reflector film^36^. As a sensor for scintillation light detection, the 8 $\times$ 8 array of SiPMs (Hamamatsu MPPC S13361-3050) was coupled with the HR-GAGG arrays and wrapped with Teflon tapes. The pitch size of the SiPM array was similar to that of the HR-GAGG array. For mechanical collimation, an 8 $\times$ 8 array of a Pb-based parallel hole collimator was equipped with an HR-GAGG pixel detector, with a hole diameter of 2 mm. The collimator thickness and pitch size were 15 mm and 3.2 mm $\times$ 3.2 mm, respectively, corresponding to those of the pixel detector.

### Signal processing and data acquisition system

The charge signals from the SiPMs were processed using the dynamic time-over-threshold (dToT) method^37,38^. The dToT method converted analog signals corresponding to the energy of gamma rays to digital signals as a time width via a comparator. Compared with the conventional signal processing method using analog-to-digital converters for pulse height measurements, the dToT method can offer lower power consumption and circuit simplicity. Thus, it was suited for multi-channel parallel signal processing. Moreover, the linearity between the time width and the radiation energy was improved by using a dynamic threshold instead of a static threshold used for the conventional ToT method^39–41^. A signal processing board comprised 64 channels of dToT-based circuits comprising an amplifier, a dynamic threshold generator, and a comparator. The intrinsic time resolution was approximately 50 ns (full width at half maximum (FWHM)). The ToT outputs were transferred to a 144-channel field-programmable gate array (FPGA, Xilinx Artix7)-based data acquisition (DAQ) system^42^ through KEL coaxial cables. The FPGA on the DAQ board operated with a clock of 500 MHz. The digital signal (T0 signal) with time information was also input to the DAQs and sent to a computer as the time data at every 1 ms interval after being processed by FPGA to synchronize the time-of-event data generated by two or more DAQs offline. Besides the time data, the event data comprising the time of flight (TOF) between the rising edge of a T0 signal and the falling edge of a ToT signal, channel number, and time width were also sent as the list mode data to the computer through the local area network. In the analysis, the time-of-event data were calculated using the time data, TOF, and time width to extract the coincidence events.

### Experimental setup

Figure 5 shows the experimental setup. Four gamma cameras were placed at 90°. A 0.5-mm-thick steel use stainless (SUS) board was placed in front of each camera to eliminate the 22 keV X-rays from ^111^In. Figure 5a-1,a-2,a-3 depict the experimental setup with the RI sources of ^111^In and ^177^Lu in 0.5 mL microtubes. Approximately 0.2 mL of ^111^InCl_3_ (Nihon medi + physics) and ^177^LuCl_3_ (POLATOM) was dispensed in each microtube (Fig. 5a-3). The radioactivities of ^111^InCl_3_ and ^177^LuCl_3_ were approximately 1.3 and 7.7 MBq, respectively. Each RI solution was colored green (^111^In) and yellow (^177^Lu). The measurement time was approximately 6 h. Two sources were placed at the center with a distance of 10 mm. Figure 5b-1,b-2,b-3 depict the experimental setup with the RI sources of ^111^In and ^177^Lu in the acrylic cases of alphabetical letters. Approximately 7.0 MBq of ^177^LuCl_3_ (< 0.2 mL) and 0.85 MBq of ^111^InCl_3_ (< 0.15 mL) were dispensed in the acrylic cases of alphabetical letters “U” and “T”, respectively (Fig. 5b-3). The width and the depth of the letters were 2 and 3 mm, respectively. The measurement time was 16 h.

### Analysis and event selection

Figure 6a illustrates the spectra of ^111^In and ^177^Lu obtained with 1 px of an HR-GAGG pixel detector. The average energy resolutions of 256 px were approximately 17.6% (FWHM) at 113 keV, 12.2% (FWHM) at 171 keV, 11.6% (FWHM) at 208 keV, and 12.0% (FWHM) at 245 keV. The energy resolution at 245 keV decreased because of the non-linearity caused by the limit of the dynamic range of the CMOS amplifier on the dToT board. Overlaps existed between the photopeaks of 171, 208, and 245 keV. The photo-absorption events of 171, 245 (from ^111^In), 113, and 208 keV (from ^177^Lu) gamma rays were extracted for imaging, which were within the time width window similar to that shown in Fig. 6b. The time width window corresponding to the energy window was decided from the calibration spectra measured without collimators. These extracted photopeak events were used for single-photon imaging, whereas the coincidence events of the cascade gamma rays within a time window were used for double-photon imaging. The $z$-positions of the extracted coincidence events were equal. Figure 6c shows the histograms of the time difference (171–245 keV for ^111^In and 113–208 keV for ^177^Lu) obtained from the experiment using microtubes. ^111^In has a relatively long duration of 85 ns at the intermediate level after emitting 171 keV gamma rays; hence, a tail caused by the duration is shown in the time difference histogram. Conversely, a tail is not shown in the time difference histogram for ^177^Lu because the 0.506 ns duration cannot be measured by our system with a 50 ns time resolution. Figure 6c indicates that the coincidence events of ^111^In and ^177^Lu were correctly detected. From these results, the time windows for ^111^In and ^177^Lu were from − 400 to 40 ns and from − 100 to 100 ns, respectively.

### Imaging method

**Figure 7:**
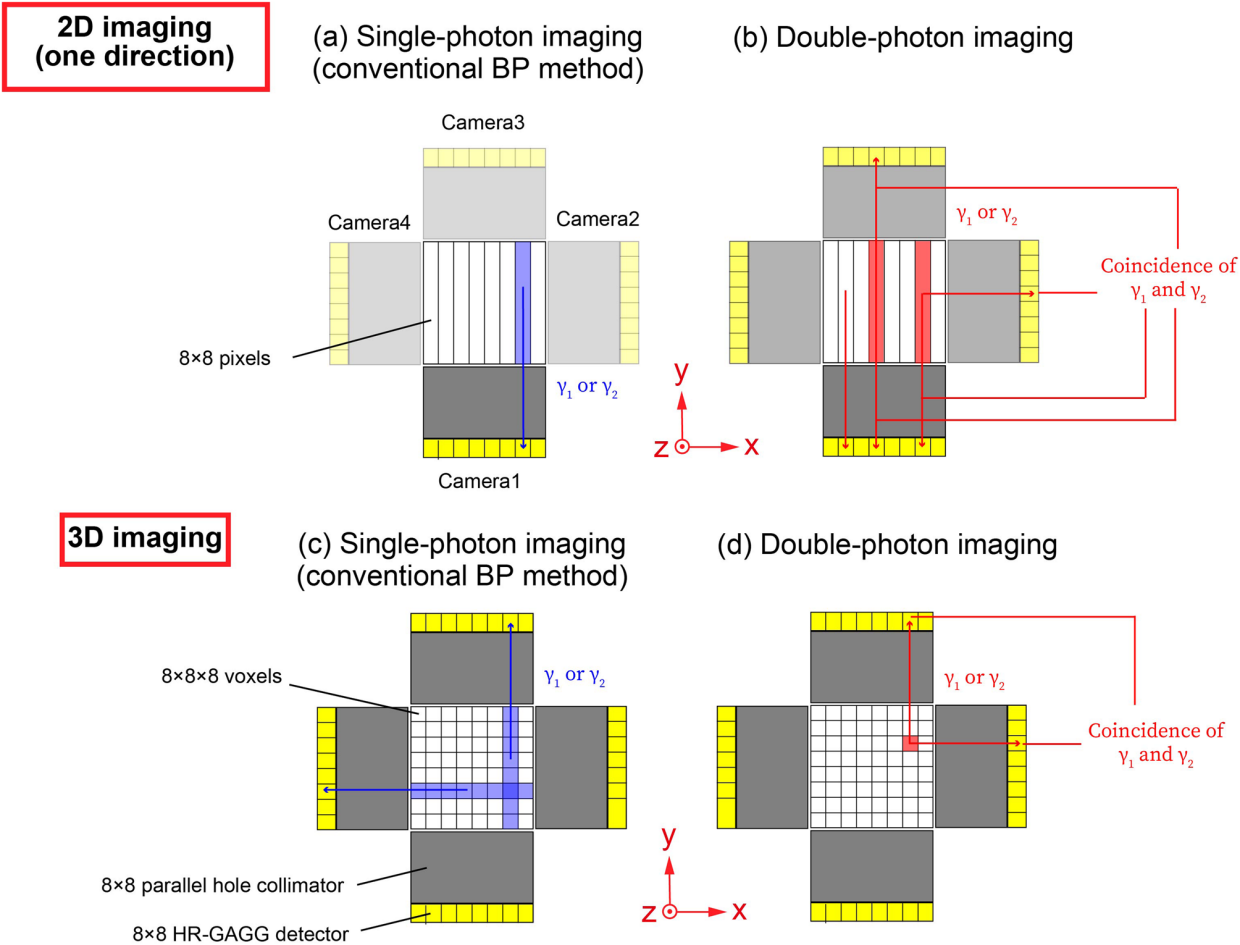
Imaging methods. (**a**) The single-photon 2D imaging method. (**b**) The double-photon 2D imaging method. The 2D imaging in one direction provides an image in the *x*–*z* plane with 8 × 8 px. The coincidence events between cameras 1 and 2, 3, or 4 are used for the double-photon coincidence 2D imaging. (**c**) The single-photon 3D imaging method. The back projection (BP) method is used for single-photon 3D imaging. Eight voxels in the *x*–*y* plane are incremented by 1/8 from two directions. (d) The double-photon 3D imaging method. The coincidence events of 90° are used for double-photon 3D imaging. A voxel determined by an intersection of the coincidence event directions is increased by 1. This figure was created using the software (Adobe Illustrator, Illustrator CC 24, https://www.adobe.com/products/illustrator.html).

Figure 7 shows the imaging method used herein. Figure 7a,b show 2D imaging methods. The photopeak events measured by Camera 1 were utilized for the single-photon 2D imaging in one direction (Fig. 7a). The coincidence events between cameras 1 and 2, 3, or 4 were used for double-photon coincidence 2D imaging (Fig. 7b). Two-dimensional imaging offers an image in the *x*–*z* plane with 8 $\times$ 8 px. An incremented pixel was determined by a detector position. In the double-photon coincidence 2D imaging, only the reduction of background was shown.

Figures 7c,d show 3D imaging methods. The BP method was applied for the single-photon 3D imaging (Fig. 7c). We did not perform the measurement with rotations; thus, eight voxels in the *x*–*y* plane were incremented by 1/8 from two directions. By contrast, the coincidence events of 90° were used for double-photon 3D imaging because these can determine the RI source position at one voxel without any imaging reconstruction method and rotation (Fig. 7d). A voxel determined by an intersection of the coincidence event directions was increased by 1. This can be expressed using the Eq. (1) like below:

4 $${\lambda }_{total}\left({\textbf{p}}_{i}^{\boldsymbol{\prime}}\right)={\sum }_{k=1}^{M}{\lambda }_{k}\left({\textbf{p}}_{i}^{\boldsymbol{\prime}}\right)$$

where ${\lambda }_{total}\left({\textbf{p}}_{i}^{\boldsymbol{\prime}}\right)$ is the value of an image voxel, ${\textbf{p}}_{i}^{\boldsymbol{\prime}}$ is the coordinate of the image voxel with the index $i$ corresponding the number of voxel, $k$ is the index corresponding to the number of 90$^\circ$ coincidence events ($k={1,2},\dots ,M)$. Then smoothing using the Gaussian function was performed for all images.

### Calculation of SBR

We evaluated the SBRs for 2D and 3D imaging of ^111^In and ^177^Lu in the microtubes. The SBR is defined as follows for the SBR evaluation of 2D imaging in one direction:

5 $$SBR = {\text{~}}\frac{{C_{{ROI}} }}{{C_{{all}} - C_{{ROI}} }},$$

where ${C}_{ROI}$ is the sum of the pixel values in the ROI (4 $\times$ 5 px) and ${C}_{all}$ is the sum of the pixel values in an image (8 $\times$ 8 px). Figure 2 presents the ROIs of the ^111^In and ^177^Lu images. The ${C}_{all}-{C}_{ROI}$ value was defined as the background.

For 3D imaging, the SBRs in the *x*- and *y*-axis were evaluated from the 1D plots of the *x*- and *y*-axis in the *x*–*y* plane. A 1D plot was extracted from the *x*–*y* plane image, including the maximum value. Figure 2a-6,a-7,b-6,b-7 depict the 1D plots normalized as 1. The background is shown as dot lines. The SBRs in the *x*- and *y*-axis are calculated as follows:

6 $$SBR = \frac{1}{{V_{b} }},$$

where ${V}_{b}$ is the background value calculated as the mean of six pixel values.
